# Hemolysis, Elevated Liver Enzymes, and Low Platelets, Severe Fetal Growth Restriction, Postpartum Subarachnoid Hemorrhage, and Craniotomy: A Rare Case Report and Systematic Review

**DOI:** 10.1155/2017/8481290

**Published:** 2017-04-16

**Authors:** Shadi Rezai, Justin Faye, Alexander Hughes, Mon-Lai Cheung, Joel R. Cohen, Judy A. Kaia, Paul N. Fuller, Cassandra E. Henderson

**Affiliations:** ^1^Department of Obstetrics and Gynecology, Southern California Kaiser Permanente, Kern County, 1200 Discovery Drive, Bakersfield, CA 93309, USA; ^2^St. George's University, School of Medicine, West Indies, Grenada; ^3^Department of Obstetrics and Gynecology, Lincoln Medical and Mental Health Center, 234 East 149th Street, Bronx, NY 10451, USA

## Abstract

*Introduction*. Hemolysis, elevated liver enzymes, and low platelets (HELLP) syndrome is a relatively uncommon but traumatic condition occurring in the later stage of pregnancy as a complication of severe preeclampsia or eclampsia. Prompt brain computed tomography (CT) or magnetic resonance imaging (MRI) and a multidisciplinary management approach are required to improve perinatal outcome.* Case*. A 37-year-old, Gravida 6, Para 1-0-4-1, Hispanic female with a history of chronic hypertension presented at 26 weeks and 6 days of gestational age. She was noted to have hemolysis, elevated liver enzymes, and low platelets (HELLP) syndrome accompanied by fetal growth restriction (FGR), during ultrasound evaluation, warranting premature delivery. The infant was delivered in stable condition suffering no permanent neurological deficit.* Conclusion*. HELLP syndrome is an uncommon and traumatic obstetric event which can lead to neurological deficits if not managed in a responsive and rapid manner. The central aggravating factor seems to be hypertension induced preeclamptic or eclamptic episode and complications thereof. The syndrome itself is manifested by hemolytic anemia, increased liver enzymes, and decreasing platelet counts with a majority of neurological defects resulting from hemorrhagic stroke or subarachnoid hemorrhage (SAH). To minimize adverse perinatal outcomes, obstetric management of this medical complication must include rapid clinical assessment, diagnostic examination, and neurosurgery consultation.

## 1. Background

The clinical syndrome of hemolysis, elevated liver enzymes, and low platelets (HELLP) is primarily coagulopathic in origin, occurring in the later stages of pregnancy after the 37th week [1]. Manifestations of HELLP can begin as epigastric pain, anemia, and platelet consumption by macroangiopathic means. The liver receives the overwhelming brunt of the coagulopathy leading to an increase in liver enzyme turnover and subsequent production [1]. HELLP syndrome is relatively uncommon, but this traumatic obstetric condition occurs at a rate of 0.1% [1]. It evolves most commonly from hypertension and can progress into severe preeclampsia or eclampsia [1–3]. HELLP syndrome can lead to cortical blindness, liver rupture, cerebral edema, hemorrhagic stroke, and subarachnoid hemorrhage [4–12]. Hemorrhagic stroke and subarachnoid brain bleeds are the most feared sequelae and the main cause of mortality from HELLP syndrome [1, 13]. Prognosis is poor in HELLP if stroke occurs in the brainstem, thalamus, and basal ganglia regions, leading to downstream ramifications with short- or long-term neurologic deficits [1, 14]. Maternal neurological deficits are lower in younger women with other risk factors evenly affecting all age groups [15, 16]. Additionally there is limited difference between strokes in pregnant and nonpregnant women [15, 16]. Prompt brain computed tomography (CT) or magnetic resonance imaging (MRI) and a multidisciplinary management approach by obstetricians and neurosurgeons are required for a favorable outcome or prognosis [1, 3, 17, 18]. HELLP, in general, provides a grim prognosis for maternal survivability while not increasing the odds of fetal mortality, which is already compromised by preeclampsia or eclamptic episodes [19].

## 2. Presentation of the Case

A 37-year-old, Gravida 6, Para 1-0-4-1, Hispanic female with advanced maternal age (AMA), history of one previous low transverse cesarean delivery 12 years ago, multiple uterine leiomyomas, chronic hypertension on Methyldopa (Aldomet) 500 mg orally, twice daily, heterozygosity for Methylenetetrahydrofolate Reductase (MTHFR) deficiency with having a 677 cytosine > thymine mutation found in 35% of Caucasian population, recurrent pregnancy loss (RPL) on Aspirin 81 mg daily and progesterone (Endometrin) 100 mg suppositories, Rh negative (s/p RhoGAM), presented at 26 weeks and 6 days of gestational age due to epigastric pain, midline upper abdominal pain, and pressure radiating to her back for one day. Obstetric history was relevant for three previous spontaneous abortions, the last occurring three years priorly, history of fetal demise at 29 weeks due to fetal gastroschisis in which patient was inducted and delivered vaginally. Physical exam showed tenderness to palpation of the epigastric region and right upper quadrant. Fetal monitoring was reassuring. Laboratory studies revealed hemolysis, elevated liver enzymes (ALT 200 IU/L, AST 209 IU/L), and low platelets 104 K/mm^3^, accompanied by severe intrauterine growth restriction (IUGR) confirmed by ultrasound and compared to previous ultrasound (3 weeks priorly). Current ultrasound correlated to a 22 weeks and 2 days of gestation, with a fetal weight of 564 grams. It is notable that the predicted mean weight is 1040 grams for an average normal fetus at 26 6/7 weeks' gestational age making our measured value less than 1st percentile [20].

The patient was placed on magnesium sulfate intravenous drip for seizure prophylaxis and fetal neuroprotection and received two doses of betamethasone for fetal lung maturity. Initial pregnancy induced hypertension (PIH) lab values showed elevated liver function tests (LFTs) with ALT of 200 IU/L and AST 209 IU/L as well as low platelets, 104 K/mm^3^. These values improved, after starting magnesium sulfate and betamethasone, to ALT of 131 IU/L, AST of 66 IU/L, and platelets 143 K/mm^3^ immediately prior to classical cesarean delivery.

Due to concomitant diagnosis of HELLP and IUGR, planned scheduled delivery after completion of second dose of steroid was made for this patient [21]. The aim was to deliver the fetus under controlled condition with maximum availability of help, including presence of multidisciplinary team of neonatal intensive care unit (NICU), respiratory therapy, and obstetrics team.

No more than 36 hours from initial presentation, the patient received morphine 0.3 mg intrathecal anesthesia and subsequently underwent repeat classical cesarean delivery at 27 weeks and 1 day for HELLP and IUGR. Her condition was further complicated by intraoperative disseminated intravascular coagulation (DIC) treated with intraoperative blood transfusion products including 2 units of packed red blood cells (PRBCs), 2 units of fresh frozen plasma (FFP), and platelets leading to a noticeable decrease in intraoperative bleeding. The patient delivered a male neonate with weight of 596 grams and APGARs of 7 and 9 in 1 minutes and 5 minutes, respectively, and was transferred to the neonatal intensive care unit (NICU) in stable condition.

On postoperative day 1 (POD 1), her DIC appeared to be resolving and lab values showed improvement: ALT 63 IU/L, AST 39 IU/L, and Plt 142 K/mm^3^. She had an unremarkable postoperative recovery and was discharged on postoperative day 2 on Methyldopa (Aldomet) 500 mg BID and a follow-up appointment in the clinic in 2 days for staple removal and blood pressure check.

On postoperative day 4, the patient returned to clinic with severe headache for 1 day, photophobia, blood soaked bandages, and an initial blood pressure of 169/78 mmHg. She denied any upper abdominal pain. The patient was immediately admitted to the inpatient service and started on magnesium sulfate drip and Methyldopa (Aldomet) 500 mg BID improving the blood pressure to 140s/80s mmHg. PIH lab values were sent (ALT 51 IU/L, AST 45 IU/L, and Plt 184 K/mm^3^) and CT scan of the head without contrast ordered to rule out any intracranial bleeding or hemorrhage.

**Figure 1:**
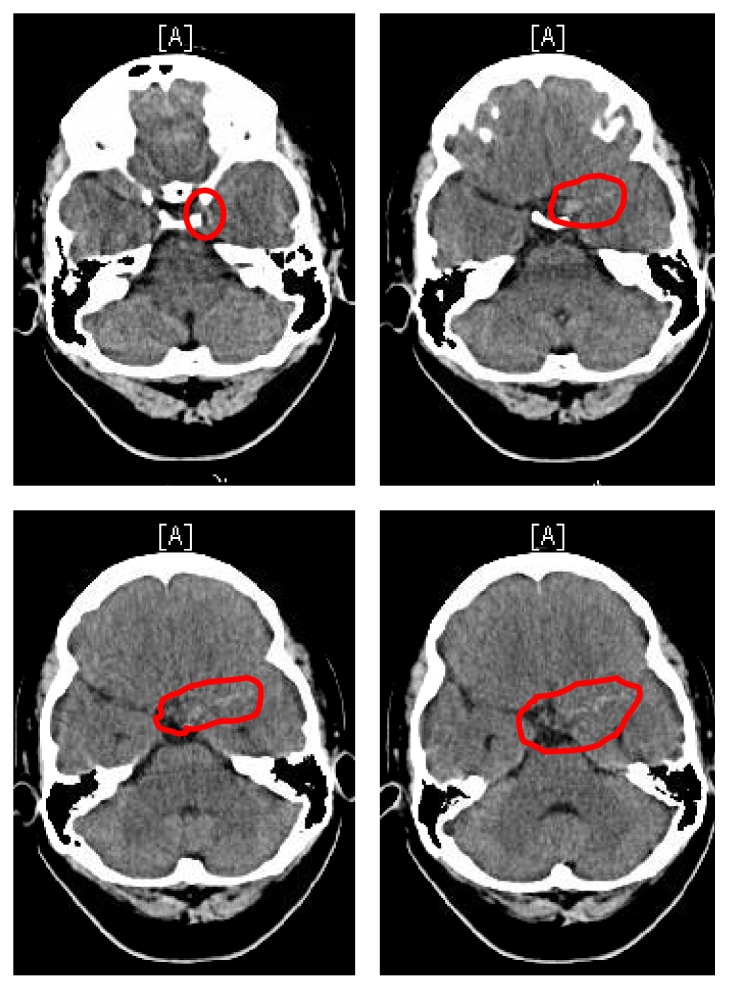
CT scan of brain without contrast axial view, on October 19, 2016, 15:51 PDT. There is mild density in the left horizontal fissure, as well as Sylvian fissure, consistent with a mild subarachnoid hemorrhage (red circles). No large acute infarction or other parenchymal lesions are identified.

CT scan of brain without contrast (please see Figure 1) showed mild density in the left horizontal fissure, as well as Sylvian fissure, consistent with a mild subarachnoid hemorrhage. An intensive care unit (ICU) consult was placed and patient immediately transferred to ICU and started on labetalol IV drip followed by nicardipine IV drip with neurosurgery consult. Further imaging by CT-angiogram, magnetic resonance angiography (MRA), and magnetic resonance venography (MRV) were inconclusive. The patient had a cerebral angiogram in the catheterization lab, which confirmed subarachnoid hemorrhage (SAH) due to a bleeding intracranial aneurysm.

**Figure 2:**
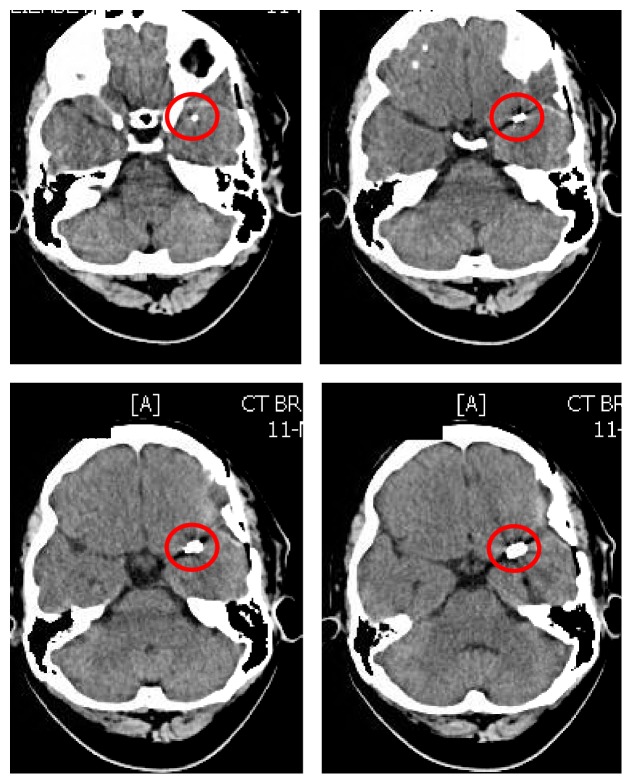
CT scan of brain without contrast axial view on November 11, 2016, after left temporal craniotomy and clipping of left MCA aneurysm: showing the clip in the area of the proximal left middle cerebral artery (red circles).

The patient was transferred to a tertiary level of care facility and underwent attempted placement of endovascular aneurysm coiling (coil embolization) by interventional radiology (IR) followed by successful left pterional/temporal craniotomy and surgical clipping of the left middle cerebral artery (MCA) aneurysm (placement of intracranial aneurysmal clip) by neurosurgery (please see Figure 2).

Following craniotomy the patient complained of intermittent headache but did not have any neurological deficits or sequelae. Follow-up blood pressure maintained a value of 130s/80s mmHg while on Aldomet. The infant was also doing well and was in stable condition in the NICU.

## 3. Discussions

Hypertension is the number one underlying cause and focus for treatment options in HELLP syndrome because it predisposes individuals to preeclampsia and subsequent HELLP syndrome [2, 3, 22–25]. Complications of HELLP syndrome range from cortical blindness, liver rupture, cerebral edema, subarachnoid hemorrhage, and most commonly hemorrhagic stroke [2, 4, 6–8, 10–12]. Most patients with hemorrhagic stroke present with headache as their main concern [9, 26–28]. A very rare complication is DIC and/or postpartum subarachnoid hemorrhage can transpire in a patient with HELLP syndrome undergoing spinal anesthesia. Though experimental, a bolus of remifentanil has shown promising effects but is limited due to the risk of neonatal depression [5, 29, 30]. The diagnosis is clinically based, with the added combination of CT visualization of increased density within regions of the brain, leading to a correlated diagnosis showing vascular brain bleeds in conjunction with neurological deficits [9, 17]. Findings can be further confirmed by MRI/MRA or IR [1]. Rapid assessment with imaging in conjunction with magnesium sulfate infusion and urgent neurosurgical decisions at a tertiary care hospital have shown positive outcomes as was seen with our patient [3, 18, 25, 31, 32].

## 4. Conclusion

HELLP syndrome is an uncommon and traumatic event which can complicate a pregnancy, leading to neurological deficits if not treated in a responsive and rapid manner. The central aggravating factor seems to be hypertension inducing a preeclamptic or eclamptic episode and complications thereof. The syndrome itself is manifested by anemia, increased liver enzymes, and decreasing platelet counts with a majority of neurological defects occurring due to hemorrhagic stroke or subarachnoid hemorrhage. Generally, HELLP syndrome provides a grim prognosis for maternal survivability, which can be improved by aggressive hypertension monitoring. Fortunately, as in this case, the incidence of maternal neurological deficits decreases in younger patients and does not increase fetal mortality which may already be compromised by preeclampsia or eclampsia.

It is important for obstetricians and nursing teams to emphasize and reemphasize the signs and symptoms of preeclampsia during prenatal visits and hospital admissions and upon discharge to all postpartum patients especially in higher risk population group with diagnosis of chronic hypertension, gestational hypertension, HELLP, preeclampsia, and eclampsia.

Educating patients on importance of close monitoring and measuring blood pressure at home, at least twice daily by the patient, and providing home blood pressure log sheet, prescription with appropriate dose of antihypertensive medications and blood pressure machine (Sphygmomanometer), and follow-up by visiting nurse services (VNS) to visit and evaluate the patient and monitor blood pressure at least twice weekly at home, as well as patient's early return visit to clinic for blood pressure check in 2-3 days following hospital discharge, all can aid in early diagnosis and address the issue of hypertension and other morbidities and mortalities associated with it.

Obstetricians should also be very cautious; although many cases of headache and elevated blood pressure in preeclamptic patients can be treated by managing patient's hypertension with antihypertensives (and addition of magnesium sulfate if indicated), supportive therapy and medications for headache, and serial neurological exam, it is important to keep in mind that intracranial hemorrhage still can be one of the etiologies for headaches in these patients which requires additional prompt brain imaging and neurosurgery evaluation.

Rapid assessment, diagnostic exams, brain imaging, and neurosurgery consultation are vital necessities for the obstetrician involved in order to provide the best long-term prognosis for mother and fetus.

## References

[1] Okada T., Kanagaki M., Yamamoto A., Fushimi Y., Togashi K. (2013). Magnetic resonance imaging of vascular encephalopathy related to pregnancy. *Neurologia Medico-Chirurgica*.

[2] Yoshikane T., Miyazaki T., Aoki S. (2013). A case of HELLP syndrome resulting in eclampsia with non-aneurysmal subarachnoid hemorrhage. *No Shinkei Geka*.

[3] Fairhall J. M., Stoodley M. A. (2009). Intracranial haemorrhage in pregnancy. *Obstetric Medicine*.

[4] Zatelli M., Comai A. (2015). Spontaneous rupture of the liver in a patient admitted for subarachnoid hemorrhage. *International Journal of Surgery Case Reports*.

[5] Koyama S., Tomimatsu T., Kanagawa T. (2010). Spinal subarachnoid hematoma following spinal anesthesia in a patient with HELLP syndrome. *International Journal of Obstetric Anesthesia*.

[6] Llovera I., Roit Z., Johnson A., Sherman L. (2005). Cortical blindness, a rare complication of pre-eclampsia. *The Journal of Emergency Medicine*.

[7] Block H. S. (2016). Neurological complications of pregnancy. *Current Neurology and Neuroscience Reports*.

[8] Aranas R. M., Prabhakaran S., Lee V. H. (2009). Posterior reversible encephalopathy syndrome associated with hemorrhage. *Neurocritical Care*.

[9] Finelli P. F. (1992). Postpartum eclampsia and subarachnoid hemorrhage. *Journal of Stroke and Cerebrovascular Diseases*.

[10] Heidrich R., Niedner K. (1970). Pregnancy and subarachnoid haemorrhage. *European Neurology*.

[11] Fliegner J. R., Hooper R. S., Kloss M. (1969). Subarachnoid haemorrhage and pregnancy. *BJOG*.

[12] Robinson J. L., Hall C. S., Sedzimir C. B. (1974). Arteriovenous malformations, aneurysms, and pregnancy. *Journal of Neurosurgery*.

[13] Lazebnik N., Pazmino R., Dierker L. R., Takaoka Y., Warf B. C. (1989). Maternal intracranial hemorrhage complicating severe superimposed preeclampsia. A case report. *Journal of Reproductive Medicine*.

[14] Miller E. C., Yaghi S., Boehme A. K., Willey J. Z., Elkind M. S., Marshall R. S. (2016). Mechanisms and outcomes of stroke during pregnancy and the postpartum period: a cross-sectional study. *Neurology: Clinical Practice*.

[15] Leffert L. R., Clancy C. R., Bateman B. T. (2015). Patient characteristics and outcomes after hemorrhagic stroke in pregnancy. *Circulation: Cardiovascular Quality and Outcomes*.

[16] Jeng J., Tang S., Yip P. (2004). Stroke in women of reproductive age: comparison between stroke related and unrelated to pregnancy. *Journal of the Neurological Sciences*.

[17] Singer J. R., Hummelgard A. B., Martin E. M. (1985). Ruptured aneurysm in pregnancy. *Journal of Neurosurgical Nursing*.

[18] Rachdi R., Fekih M. A., Massoudi L. (1993). HELLP syndrome. Epidemiological, nosological and prognostic aspects. *Revue Francaise de Gynecologie et d'Obstetrique*.

[19] Hadlock F. P., Harrist R. B., Martinez-Poyer J. (1991). In utero analysis of fetal growth: a sonographic weight standard. *Radiology*.

[20] Vilela P., Duarte J., Goulão A. (2001). Cerebrovascular disease in pregnancy and puerperium. *Acta Médica Portuguesa*.

[21] Bienstock J. L., Fox H. E., Wallach E. E. (2015). *The Johns Hopkins Manual of Gynecology and Obstetrics*.

[22] Bushnell C., McCullough L. (2014). Stroke prevention in women: synopsis of the 2014 American heart association/american stroke association guideline. *Annals of Internal Medicine*.

[23] Li R., Mitchell P., Dowling R., Yan B. (2013). Is hypertension predictive of clinical recurrence in posterior reversible encephalopathy syndrome?. *Journal of Clinical Neuroscience*.

[24] Tanguay J. J., Allegretti P. J. (2008). Postpartum intracranial hemorrhage disguised as preeclampsia. *The American Journal of Emergency Medicine*.

[25] Mathew M., Salahuddin A., Mathew N. R., Nandhagopal R. (2016). Idiopathic intracranial hypertension presenting as postpartum headache. *Neurosciences*.

[26] Wiles K. S., Nortley R., Siddiqui A., Holmes P., Nelson-Piercy C. (2015). Reversible cerebral vasoconstriction syndrome: a rare cause of postpartum headache. *Practical Neurology*.

[27] Farine D., Andreyko J., Lysikiewicz A., Simha S., Addison A. (1984). Isolated angiitis of brain in pregnancy and puerperium. *Obstetrics and Gynecology*.

[28] Palacio F., Ortiz-Gómez J., Fornet I., López M., Morillas P. (2008). Remifentanil bolus for cesarean section in high-risk patients: study of 12 cases. *Revista Española de Anestesiología y Reanimación*.

[29] Roberts J. M., May W. J. (1976). Consumptive coagulopathy in severe preeclampsia. *Obstetrics & Gynecology*.

[30] Brouh Y., Jean K. K., Ouattara A. (2016). Brain lesions in eclampsia: a series of 39 cases admitted in an Intensive Care Unit. *Indian Journal of Critical Care Medicine*.

[31] Wong G. K., Chan M. T., Gin T., Poon W. S. (2011). Intravenous magnesium sulfate after aneurysmal subarachnoid hemorrhage: current status. *Acta Neurochirurgica Supplement*.

[32] Brewer R. P., Parra A., Lynch J., Chilukuri V., Borel C. O. (2001). Cerebral blood flow velocity response to magnesium sulfate in patients after subarachnoid hemorrhage. *Journal of Neurosurgical Anesthesiology*.

